# Authors' Comment on “Brain Tumours: Rise in Glioblastoma Multiforme Incidence in England 1995–2015 Suggests an Adverse Environmental or Lifestyle Factor”

**DOI:** 10.1155/2018/2170208

**Published:** 2018-06-25

**Authors:** Alasdair Philips, Denis L. Henshaw, Graham Lamburn, Michael J. O'Carroll

**Affiliations:** ^1^Children with Cancer UK, 51 Great Ormond Street, London WC1N 3JQ, UK; ^2^Powerwatch, Cambridgeshire, UK; ^3^University of Bristol, Bristol, UK; ^4^Vice-Chancellor's Office, University of Sunderland, Sunderland, UK

At the request of the Editor, we here provide further background to our article [1].

Over the past 20 years, the authors have been involved in organising international conferences on causes of cancer and finding precautionary actions that may help to reduce the ongoing overall rise in the cancer burden [2, 3]. As part of this, we follow trends in cancer incidence across all ages. We were hearing from clinicians that they were seeing an increase in aggressive brain tumours, especially glioblastoma multiforme (GBM), but cancer registries were generally reporting no significant overall increased incidence in brain tumours.

By 2008, we were seeing a statistically significant increased incidence in frontal and temporal lobe tumours and a decrease in tumour incidence at some other brain sites listed in UK Office for National Statistics (ONS) MB1 cancer data. In November 2011, three of the current authors discussed this rise during a two-day EC conference in Brussels. We were told by two leading European epidemiologists that if we could not see a clear trend in the overall data, there was no point in looking at underlying, more detailed data.

The rising incidence in frontal and temporal lobes continued to appear in the public MB1 data and we decided to formally test our suspicion that something important was changing. We applied for, and obtained, more detailed information from the ONS which included ICD-O-10 coding. As part of our ongoing monitoring, we have twice had these data updated. This resulted in our current article [1], where we report a clear increasing trend in GBM incidence over time. In the article, we also briefly discuss a number of different possible causal factors that have been reported in the scientific literature.

We acknowledge that published data from the US Central Brain Tumor Registry (CBTRUS) and the Surveillance, Epidemiology, and End Results (SEER) organisations do not report a similar rise in GBM. One factor will be that, according to CBTRUS, GBM incidence rate in black people is approximately half that for white people and has a different age-related profile [4]. The US has a higher percentage of black people compared with England and this will have some effect on the whole-population brain tumour data profile.

However, we have come to an initial conclusion that the main reason is due to (a) the US2000 Standard Population that they use to adjust their data and (b) the fact that they use age-standardised data even for age-grouped data that would usually be age-specific. Age-standardised data are used for comparing overall rates between countries and for following overall data trends within a country.

All the data in the CBTRUS reports (see Figure 13 in each reference [5, 6]) are stated as being adjusted to the US2000 Standard Population [7]. This does not reasonably represent the age spectrum of the current US population. This is shown in Figure 1.

The current US population is very different from the US2000 Standard Population. The effect of applying US2000 is to reduce, by about 30%, the overall contribution from cases in people aged 50 to 70 years. This is the age range of the majority of the cases in the ONS data which show the rising GBM incidence trend. Using US2000 gives added weight to the “healthy worker” age range (30 to 44), where relatively few GBM cases occur. It is important that the age standardisation profile is a reasonable fit to the current population age profile. We note that SEER updated the US Standard Population every ten years from 1940 to 2000 but have not done so since 2000 [6].

We offer, for discussion, Figure 2, where we have back-adjusted the US age-group data from US2000 to the actual US population data for 2008 and 2012 [8], along with English ONS data for two five-year periods from our article. The readjusted US data now show an increase in GBM similar to our findings. US data of unadjusted, age-specific GBM incidence rates for all 5-year age groups for every year from 2005 and 2015 are required to check our approximate correction.

We would like to add to the discussion of potential risk factors in our article.

There is a growing body of evidence that exposure to air pollution, notably arising from the carcinogenic components of vehicle exhausts, such as PAHs, 1,3-butadiene, and diesel particulate matter generally, may be associated with increased risk of brain tumours in both children and adults. Studies in children, young adults, and canines indicate that inhaled ultrafine air pollution particles, ~100 nm, pass through the lung to reach and both damage and cross the blood-brain barrier (Calderón-Garcidueñas et al. (2008) [9], (2003) [10]).

Braüner et al. (2013) [11] reported an association between calculated domestic radon exposure and brain tumour incidence in a Danish cohort, with doubling of risk with each 100 Bq.m^−3^ increment in average residential radon levels. Indoor radon levels have increased in recent decades with the progressive introduction of double-glazed windows and general house sealing, resulting in lower rates of air changes with outside air.

Ostrom et al. [12] considered mobile phone use and judged that the current evidence* “was inconclusive”* but recommended continued monitoring of this issue. De Vocht (2016) [13] concluded that* “A causal factor, of which mobile phone use (and possibly other wireless equipment) is in agreement with the hypothesized temporal association, is related to an increased risk of developing malignant neoplasms in the temporal lobe.”* He later published an important correction to the article, showing a large rise in GBM tumour incidence with time [14]. The article reports that the GBM rise was not associated with his mobile phone use impact modelling, but we note that the model was only for primary initiation and not promotion of lower-grade tumours.

Our article does not focus on any particular risk factor to explain the rising incidence of aggressive GBM tumours, which are usually quickly fatal. We recommend that our detailed analyses be repeated for cancer registry data in other countries. If our results are confirmed, then high priority should be given to identifying the factors involved in the rise.

## Figures and Tables

**Figure 1:**
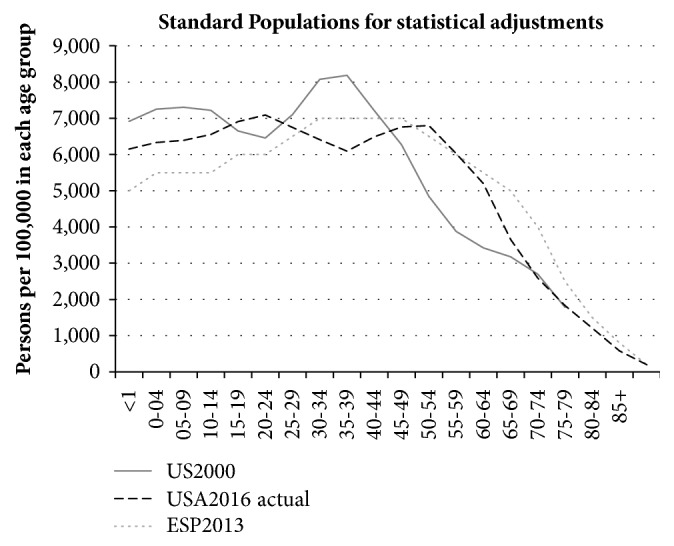
US2000 Standard Population, the 2016 US actual population, and ESP2013.

**Figure 2:**
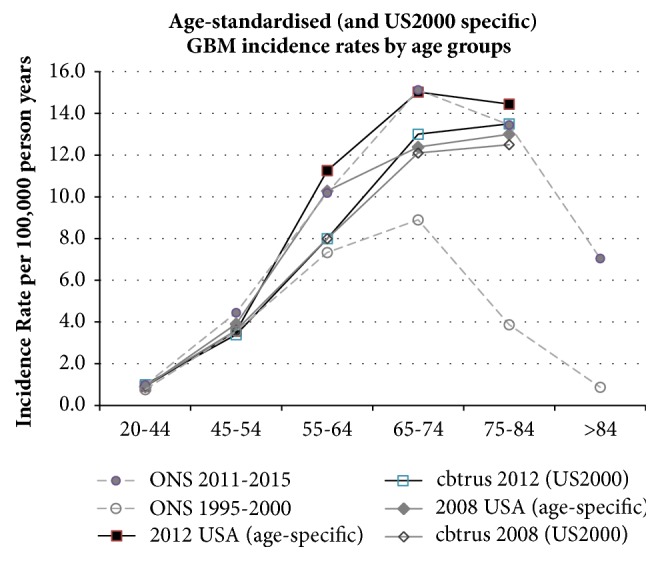
Comparison of ONS and US data trends following adjustment to age-specific rate.
